# Physiological levels of nitrate support anoxic growth by denitrification of *Pseudomonas aeruginosa* at growth rates reported in cystic fibrosis lungs and sputum

**DOI:** 10.3389/fmicb.2014.00554

**Published:** 2014-10-24

**Authors:** Laura Line, Morten Alhede, Mette Kolpen, Michael Kühl, Oana Ciofu, Thomas Bjarnsholt, Claus Moser, Masanori Toyofuku, Nobuhiko Nomura, Niels Høiby, Peter Ø. Jensen

**Affiliations:** ^1^Department of Clinical MicrobiologyRigshospitalet, Copenhagen, Denmark; ^2^Department of International Health, Immunology and Microbiology, Faculty of Health Sciences, University of CopenhagenCopenhagen, Denmark; ^3^Marine Biological Section, Department of Biology, University of CopenhagenCopenhagen, Denmark; ^4^Plant Functional Biology and Climate Change Cluster, University of Technology SydneySydney, NSW, Australia; ^5^Singapore Centre on Environmental Life Sciences Engineering, Nanyang Technological UniversitySingapore, Singapore; ^6^Graduate School of Life and Environmental Sciences, University of TsukubaTsukuba, Japan

**Keywords:** *Pseudomonas aeruginosa*, denitrification, growth, cystic fibrosis

## Abstract

Chronic *Pseudomonas aeruginosa* lung infection is the most severe complication in patients with cystic fibrosis (CF). The infection is characterized by the formation of biofilm surrounded by numerous polymorphonuclear leukocytes (PMNs) and strong O_2_ depletion in the endobronchial mucus. We have reported that O_2_ is mainly consumed by the activated PMNs, while O_2_ consumption by aerobic respiration is diminutive and nitrous oxide (N_2_O) is produced in infected CF sputum. This suggests that the reported growth rates of *P. aeruginosa* in lungs and sputum may result from anaerobic respiration using denitrification. The growth rate of *P. aeruginosa* achieved by denitrification at physiological levels (~400 μM) of nitrate (NO^−^_3_) is however, not known. Therefore, we have measured growth rates of anoxic cultures of PAO1 and clinical isolates (*n* = 12) in LB media supplemented with NO^−^_3_ and found a significant increase of growth when supplementing PAO1 and clinical isolates with ≥150 μM NO^−^_3_ and 100 μM NO^−^_3_, respectively. An essential contribution to growth by denitrification was demonstrated by the inability to establish a significantly increased growth rate by a denitrification deficient Δ*nirS-N* mutant at <1 mM of NO^−^_3_. Activation of denitrification could be achieved by supplementation with as little as 62.5 μM of NO^−^_3_ according to the significant production of N_2_O by the nitrous oxide reductase deficient Δ*nosZ* mutant. Studies of the promoter activity, gene transcripts, and enzyme activity of the four N-oxide reductases in PAO1 (Nar, Nir, Nor, Nos) further verified the engagement of denitrification, showing a transient increase in activation and expression and rapid consumption of NO^−^_3_ followed by a transient increase of NO^−^_2_. Growth rates obtained by denitrification in this study were comparable to our reported growth rates in the majority of *P. aeruginosa* cells in CF lungs and sputum. Thus, we have demonstrated that denitrification is required for *P. aeruginosa* growth in infected endobronchial CF mucus.

## Introduction

Chronic lung infection by *Pseudomonas aeruginosa* is the most severe complication in cystic fibrosis (CF) patients (Koch and Høiby, 2000). The infection is characterized by biofilm aggregates of *P. aeruginosa* within the endobronchial mucus surrounded by high densities of active polymorphonuclear leukocytes (PMNs) (Bjarnsholt et al., 2009). The activity of PMNs has been shown to be the major cause of O_2_ depletion due to production of the reactive oxygen species superoxide (O^−^_2_) and nitric oxide (NO) within sputum samples (Kolpen et al., 2010, 2014a). Combined with the O_2_ consumption by lung epithelial cells, micro-oxic to anoxic conditions can develop in the airways of CF patients (Worlitzsch et al., 2002), which have been further evidenced by the upregulation of the O_2_ sensing anaerobic global regulator (Anr) in *P. aeruginosa* isolates (Hoboth et al., 2009) and the presence of obligate anaerobes such as *Prevotella* within sputum (Tunney et al., 2008). While O_2_ depletion by microbial aerobic respiration is thus diminutive, *P. aeruginosa* has been shown to be actively growing within the mucus of lungs (Kragh et al., 2014) and sputum (Yang et al., 2008). As a facultative anaerobe, this growth may be attributable to *P. aeruginosa's* ability to perform anaerobic respiration by denitrification.

**Figure 1:**
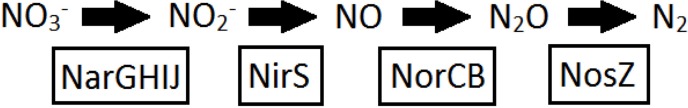
**Denitrification pathway**. Reduction of nitrate to nitrite, nitric oxide, nitrous oxide, and finally dinitrogen. The four reductase enzymes nitrate reductase, nitrite reductase, nitric oxide reductase, and nitrous oxide reduction are boxed below. Nitrite reduction marks the first step of strict denitrification.

Denitrification was first described as the liberation of gaseous nitrogen oxides (N-oxides) by bacteria (Gayon and Dupetit, 1886) and involves the utilization of N-oxides as alternative electron acceptors for respiration (Figure 1). This definition of denitrification means that while nitrate (NO^−^_3_) reduction to nitrite (NO^−^_2_) contributes directly to energy generation via the proton pumping action of the membrane-bound nitrate reductase (NarGHIJ) (Chen and Strous, 2013), the first step in strict denitrification is actually NO^−^_2_ reduction, where NO is the first gaseous product arising from NO^−^_3_ reduction (Zumft, 2005).

Engagement of denitrification in infected CF lungs is in accordance with the production of the denitrification intermediate nitrous oxide (N_2_O) in sputum (Kolpen et al., 2014b), the presence of the denitrification marker OprF and antibodies against OprF in lung secretions and sera (Yoon et al., 2002), antibodies against Nar in sera (Beckmann et al., 2005) and the upregulation of the denitrification reductases in CF isolates (Son et al., 2007; Hoboth et al., 2009; Lee et al., 2011). Additionally, after antimicrobial treatment the infected sputum content of NO^−^_3_ increases (Grasemann et al., 1998) indicating a reduction in the activity of denitrifying cells.

## Materials and methods

### Bacterial strains and plasmids

The strains and plasmids used in this study are listed in Table 1. Primers used in this study are listed in Table 2. The wild type strain used for experiments and reporter plasmid insertion was PAO1 obtained from the Pseudomonas Genetic Stock Center (www.pseudomonas.med.ecu.edu, strain PAO0001). The Holloway PAO1 wild type was used as the control for the examinations of the Holloway PAO1 Δ*nirS-N* and the Holloway PAO1 Δ*nosZ* mutants. The PAO1 strains grew at similar rates under anoxic conditions.

**Table 1:** ***P. aeruginosa* strains used for this study**.

**Strains and plasmids**	**Details**	**Study**
**PLASMIDS**		
pMEXGFP	pMEX9 derived promoter-probe vector; *egfp* Gm^r^	Toyofuku et al., 2013
pMEXdsREDex	pMEX9 derived promoter-probe vector; *dsredexpress* Gm^r^	This study
pMNarK_1_-GFP	*narK_1_* promoter region fused to *egfp* in pMEXGFP	This study
pMNirS-dsREDex	*nirS* promoter region fused to *dsredexpress* in pMEXdsREDex	This study
pMNorC-GFP	*norC* promoter region fused to *egfp* in PMEXGFP	This study
pMNosR-dsREDex	*nosR* promter region fused to *dsredexpress* in pMEXdsREDex	This study
pET15b	Expression vector Ap^r^	Novagen, Madison, WI, USA
pG19*nir*	*nirS to nirN* deletion cassette in PG19II	Toyofuku et al., 2013
pG19*nos*	*nosZ* deletion cassette in PG19II	This study
**STRAINS**		
*E. coli*		
S17-1	Mobilizer strain	Simon et al., 1986
*P. aeruginosa*		
PAO1 MH340	WT	Pseudomonas Genetic Stock Center
PAO1 *egfp*	Background control	Toyofuku et al., 2013
PAO1 *dsredexpress*	Background control	This study
PAO1 *narK_1_-eGFP*	Nitrate reductase reporter	This study
PAO1 *nirS-dsredexpress*	Nitrite reductase reporter	This study
PAO1 *norC-eGFP*	Nitric oxide reductase reporter	This study
PAO1 *nosR-dsredexpress*	Nitrous oxide reductase reporter	This study
Holloway PAO1	WT	Holloway et al., 1979
Holloway Δ*nirS-N*	*nirS* to *nirN* deleted mutant	Toyofuku et al., 2013
Holloway Δ*nosZ*	*nosZ* deleted mutant	This study
I M/NM	Clinical isolate	Kolpen et al., 2014c
II M/NM	Clinical isolate	Kolpen et al., 2014c
III M/NM	Clinical isolate	Kolpen et al., 2014c
IV M/NM	Clinical isolate	Kolpen et al., 2014c
V M/NM	Clinical isolate	Kolpen et al., 2014c
VI NM	Clinical isolate	Kolpen et al., 2014c
VII NM	Clinical isolate	Kolpen et al., 2014c

*Gm^r^ (Gentamicin resistant), Ap^r^ (Ampicillin resistant), Mucoid (M), Non-mucoid (NM), Wild type (WT)*.

**Table 2:** **Primer sequences used in this study**.

**Primers**	**Sequence**	**Study**
**REPORTER PLASMIDS**		
Gfp F1	5′ – ACGCCAAGCTTGCATGCCTG – 3′	Toyofuku et al., 2013
pET15b-kpn	5′ – GGGGTACCACTTTAAGAAGGAGATATACCATGG – 3′	Toyofuku et al., 2013
dsred R1	5′ – GGGCTCGAGCTACAGGAACAGGTGGTGGCGGC – 3′	This study
dsred R2	5′ – CGAGCTCCTACAGGAACAGGTGGTGGCGGC – 3′	This study
pNarK_1_ F	5′ – GGAATTCCCGGCGTGGTTGATCACCGC – 3′	This study
pNarK_1_ R	5′ – TTCAAGCTTAGGCCAGGCCGTAGATCGGG – 3′	This study
pNirS F	5′ – CCCAAGCTTCTTTCATGTCGTCCTTGGCGTG – 3′	This study
pNirS R	5′ – CCCAAGCTTGCCCTTGAGCAATACCGGCAG – 3′	This study
pNorC F	5′ – CGGAATTCGCGAGCGCTTCATGGAGCTGG – 3′	This study
pNorC R	5′ – GCCAAGCTTGCCGAAATAGATGTTCCTGGCC – 3′	This study
pNosR F	5′ – CGGGATCCCCTGGTACCGTTACCTGAAGGC – 3′	This study
pNosR R	5′ – CCCAAGCTTGGATCACCTGCAGGTTGATCGG – 3′	This study
**MUTANTS**		
Δ*nirS-N* F1	5′ – CGGAATTCCCAGTAGAGGGTGAAGAAAGTGTCGTGC – 3′	Toyofuku et al., 2013
Δ*nirS-N* R1	5′ – CGGGATCCGACCGGAATCAAGATTGCGTTGCTTTGC – 3′	Toyofuku et al., 2013
Δ*nirS*-*N* F2	5′ – CGGGATCCGGTCGAGACGCCATCAACGGATATCC – 3′	Toyofuku et al., 2013
Δ*nirS-N* R2	5′ – CCGAAGCTTGCGATGTTCACCATGATGAACTACGAGC – 3′	Toyofuku et al., 2013
Δ*nosZ* F1	5′ – CGCAAGCTTGGGTCTGGTACCAGAAGAGTTTCCAGG – 3′	This study
Δ*nosZ* R1	5′ – GACTAGTGTCGCTCATCTCGTGTCGTCCTCAAGGG – 3′	This study
Δ*nosZ* F2	5′ – GACTAGTCCGCAACAGACCTCGTCGATCACC – 3′	This study
Δ*nosZ* R2	5′ – CGCAAGCTTCGAGTAGGTGATGTAGTTCATCAGGATGCC – 3′	This study
**qPCR**		
*narK_1_*F	5′ – CTCCTCCTGCTTCATGTTGCT – 3′	This study
*narK_1_*R	5′ – TGCTGCTTGATCGCGTAGTG – 3′	This study
*nirS* F	5′ – GGCGGCTCGCTGTTCAT – 3′	This study
*nirS* R	5′ – TGAAGGTGGTGTCGACGTAGAG – 3′	This study
*norC* F	5′ – AAGGCATGGCCAGGAACAT – 3′	This study
*norC* R	5′ – CCAGGAACAGCAGGATGAAGA – 3′	This study
*nosZ* F	5′ – CGCTGCACACCACTTTCG – 3′	This study
*nosZ* R	5′ – CACCAACTGGCTGTCGATGA – 3′	This study
*rpoD* F	5′ – ACAAGATCCGCAAGGTACTGAAG –3′	This study
*rpoD* R	5′ – CGCCCAGGTGCGAATC – 3′	This study

*Forward (F), Reverse (R)*.

Plasmids containing eGFP or DsRed-Express tagged reporter genes were derived from pME as pG19nir and pG19nos plasmids carrying deletion cassettes of *nirS* to *nirN* and *nosZ* were constructed with the same procedure described previously (Maseda et al., 2004). The pG19II-derived plasmids were transferred into Holloway PAO1 by conjugating with *E. coli* S17-1 (Simon et al., 1986) followed by homologous recombination described elsewhere (Maseda et al., 2004). The mutants were analyzed by polymerase chain reaction (PCR) (Toyofuku et al., 2013).

The eGFP-based promoter probe plasmid, pMEXGFP, was constructed as described previously (Toyofuku et al., 2013). The promoter regions of *narK_1_* and *norC* were amplified with pNarK_1_ F/pNarK_1_ R or pNorC F/pNorC R primer pairs and cloned into pMEXeGFP for promoter activity assays. pMEXdsREDex was constructed in the same way, with *dsredexpress* amplified using the primer pairs Gfp F1/dsred R1 (Table 2) and post subcloning, the primer pair pET15b-kpn/dsred R2 (Table 2) was used to amplify a RBS-fused DsRed-Express to clone into the pMEX9 plasmid (Toyofuku et al., 2007). The promoter regions of *nirS* and *nosR* were amplified with the primer pairs pNirS F/pNirS R or pNosR F/pNosR R (Table 2) and cloned into the pMEdsREDex for promoter activity assays.

The reporter plasmid insertion was achieved by preparation of electro-competent cells as previously described (Choi et al., 2005) and subsequent electroporation at 25 μF, 200 Ω, 2.5 kV with 1 μl of plasmid DNA for 100 μl of electro-competent cells. Transformed cells were selected for by plating with 30 ng ml^−1^ gentamicin (Sigma-Aldrich, St. Louis, MO, USA) and frozen cultures were produced from a single colony.

Clinical strains (5 mucoid and 7 non-mucoid) were isolated from expectorated sputum of 7 chronically infected CF patients (Kolpen et al., 2014b).

### Anoxic growth experiments

In this study, we first establish experimental evidence for the ability of physiological levels of NO^−^_3_ to support anoxic growth of *P. aeruginosa* PAO1 wild type and 12 clinical isolates at rates comparable to those in CF lungs and sputum. To discern that a significant portion of the attained growth was attributable to denitrification, a strain incapable of denitrification due to a mutation in the nitrite reductase (Nir) operon was studied.

All experiments requiring anoxic conditions were performed in an anoxic bench (Concept 400, Thermo Scientific, Waltham, MA, USA) with O_2_ <0.02% routinely monitored with an luminescent dissolved oxygen O_2_ sensor (HQ40d multi, HACH Company, Loveland, CO, US). Media was sealed with parafilm (Bemis, Neenah, WI, USA) and allowed to equilibrate for a minimum of 3 days. Strains were plated from frozen cultures and single colonies were used to initiate normoxic overnight cultures. They were then adjusted to OD_600_ 0.1 and regrown to OD_600_ 0.4 to ensure all cells were in the exponential growth phase. The culture was inoculated by diluting 1:100 in relevant anoxic Luria-Bertani broth (LB) media supplemented with KNO_3_ (Sigma-Aldrich) to obtain the noted concentrations of NO^−^_3_ in 2 ml glass vials (Schuett Biotec, Göttingen, DE), which were sealed inside the anoxic bench before transfer to shaking at 200 rpm. Physiological levels of NO^−^_3_ here are considered to be approximately 400 μM with a maximum of 1 mM based on the concentrations of NO^−^_3_ detected in CF sputum (Grasemann et al., 1998; Palmer et al., 2007; Kolpen et al., 2014a). Vials were shown to remain anoxic for a minimum of 2 days using anaerobic indicator strips (SSI Diagnostica, Hillerød, Denmark) that changed from white to blue in the presence of oxygen. Optical density was measured directly in the vials at 600 nm (OD_600_) by a spectrophotometer (Shimadzu, Kyoto, JP) and values obtained were shown to correlate linearly to dilutions of an overnight culture in LB at OD_600_ values > 0.02. Accordingly, only OD values higher than 0.02 were used in growth rate calculations. Growth rates were calculated as described (Widdel, 2007).

The suitability of the assay was demonstrated in cultures of PAO1 supplemented with 100 mM NO^−^_3_, where our measured growth rate of 0.7 divisions h^−1^ and final OD_600_ of 1.0 (data not shown) corresponded well with previously reported growth rates of *P. aeruginosa* (Alvarez-Ortega and Harwood, 2007; Lee et al., 2012; Fang et al., 2013).

### Promoter activity—reporter strains

To further confirm the engagement of denitrification, the transcripts and promoter activity of the four N-oxide reductases (Nar, Nir, Nor, Nos) were studied using qRT-PCR and fluorescent reporter strains, respectively. Reporter strains with the promoter region of the four denitrification reductase operons; *narK_1_K_2_GHIJ, nirSMCFDLGHJEN, norCBD, nosRZDFYL* (Schobert and Jahn, 2010) fused to fluorescent tags of either eGFP or DsRed-Express (Table 1) were used to study promoter activation under anoxic conditions with 400 μM KNO_3_ supplemented LB as described above.

GFP and DsRed require O_2_ for chromophore maturation (Craggs, 2009; Strack et al., 2010). Consequently, 1600 ng ml^−1^ chloramphenicol (Sigma-Aldrich) was added to the cultures to prevent new protein synthesis (Toyofuku et al., 2013) with shaking for 1 h at 37°C in atmospheric air prior to measurement to allow the chromophore to develop—termed oxic fluorescence recovery (OFR) (Zhang et al., 2005). The signal was detected by flow cytometry as described below. The fluorescence values obtained were corrected for background fluorescence seen in the control strains with *eGFP* and *dsredexpress* unconjugated to a promoter.

### Flow cytometry

Detection of reporter strain fluorescence was performed using a FACSCanto flowcytometer (BD Biosciences, San Jose, CA, USA) with a 488 nm argon laser. eGFP was detected in FL-1 with a 502 nm long pass filter and a 530/30 nm band pass emission filter. DsRed-Express fluorescence was collected in FL-2 with a 556 nm long pass filter and 585/42 nm band pass emission filter. To maximize resolution, samples were analyzed at low flow rate corresponding to 10 μl min^−1^. At least 10,000 events were recorded for each sample. Cytometer Setup and Tracking Beads (BD Biosciences) were used for instrument calibration, and flow data were processed and analyzed by Diva (BD Biosciences).

### Denitrification gene transcript expression—RNA extraction and reverse transcription

PAO1 was grown in triplicate for 3 h under anoxia with either unsupplemented LB or LB with 400 μM KNO_3_. The 3 h time point was chosen as it represented the approximate peak of promoter activity, as seen in the reporter strains. After 3 h, the cultures were mixed with 2 volumes of RNA*later* (Ambion, Austin, TX, USA) within the anoxic bench for transcript stabilization and kept at 4°C overnight before freezing at −80°C. The cultures were subsequently thawed on ice, centrifuged to harvest the cells and lysed with 100 μl of 1 mg ml^−1^ lysozyme (Affymetrix, Cleveland, OH, USA) at room temperature for 13 min. Total RNA was extracted with RNeasy Mini Kits (Qiagen Danmark, Limburg, NL) and contaminating chromosomal DNA was removed by treatment with RQ1 RNase-free DNase (Promega, Madison, WI, USA). RNA quality was detected in a NanoDrop spectrophotometer (Thermo Scientific). cDNA was synthesized from 250 to 500 ng of RNA (depending on the concentration extracted) and High Capacity RNA-to-cDNA Master Mix (Applied Biosystems, Foster City, CA, US) with a final reaction volume of 20 μl in a 2720 Thermal Cycler (Applied Biosystems). The conditions consisted of 5 min at 25°C, 30 min at 42°C, 5 min at 85°C and subsequent holding at 4°C. cDNA was frozen at −20°C. Negative controls contained template with the Master Mix replaced with water. cDNA was diluted 1:100 for qPCR.

### Primer design

Primers for the denitrification genes *narK_1_, nirS, norC, nosR* and the reference gene *rpoD* were designed using Integrated DNA Technologies Primer Quest software (http://www.idtdna.com) using sequences from the PAO1 Genome Sequence, *Pseudomonas* Genome Database (Winsor et al., 2011) (Table 2).

### Quantitative PCR

Each PCR mix had a final volume of 20 μl containing 10 μl SYBR Green qPCR Master Mix 2x (Applied Biosystems) 2 μl cDNA, 2 μl of 2 pmol μl^−1^ forward and reverse primers and 4 μl water. Quantitative real-time PCR was performed with a StepOnePlus, Real-Time PCR system (Applied Biosystems) with initial denaturation at 95°C for 10 min followed by 40 cycles of 95°C for 15 s, 60°C for 30 s and 72°C for 30 s.

Data were normalized to the expression of *rpoD*, a housekeeping gene encoding the primary sigma factor for RNA polymerase found to be constitutively expressed in *P. aeruginosa* (Savli et al., 2003). To control for variations between runs, the housekeeping and target genes for each of the three samples were amplified in one 96-well plate. Expression of the target genes in the cultures with 400 μM KNO_3_ was compared to the unsupplemented cultures and the difference expressed as n-fold change.

### NO^−^_3_ and NO^−^_2_ quantification

For measuring NO^−^_3_ and NO^−^_2_ concentration in the culture media, the supernatant was harvested and sterile filtrated through a syringe filter (TRP, Trasadingen, SUI) (pore size 0.22 μm) and frozen at −20°C for later measurement. NO^−^_3_ and NO^−^_2_ were detected in 96-well microtiter plates using a modified Griess nitrite/nitrate colorimetric assay (Cayman Chemicals, Ann Arbor, MI, USA) according to manufacturer's instructions and as previously described (Kolpen et al., 2014a). Briefly, NO^−^_2_ concentration was estimated by incubating with the Griess Reagent, converting NO^−^_2_ into a purple azo-compound detected by absorbance at 540 nm in a Multiskan EX plate reader (Thermo Scientific). For NO^−^_3_ concentration, NO^−^_3_ was first converted to NO^−^_2_ by incubation with NO^−^_3_ reductase and subsequent addition of the Griess reagent to detect total NO^−^_3_ plus NO^−^_2_. The NO^−^_3_ concentration was calculated as the difference between the total NO^−^_2_ and NO^−^_3_ concentration and the NO^−^_2_ concentration. The detection limit of the assay was ~2 μM for NO^−^_2_ and ~5 μM for NO^−^_3_.

### Microsensor measurements of N_2_O

Additionally, reductase activity was elucidated by measuring consumption of NO^−^_3_ and production of NO^−^_2_ as well as measurements of N_2_O gas accumulation in a Δ*nosZ* mutant strain incapable of the final reduction step in denitrification of N_2_O to N_2_. Each small glass vial (Schuett Biotec, Göttingen, DE) containing anoxic planktonic cultures of either PAO1 WT or the Δ*nosZ* mutant were placed in a 37°C heated rack and kept sealed prior to individual measurements, upon which they were shaken and the lid removed. Immediately after, N_2_O was detected in the media using an amperometric N_2_O microsensor (Andersen et al., 2001) with a tip diameter of 25 μm (Unisense A/S, Aarhus, DK) as previously described (Kolpen et al., 2014a). Each measurement consisted of a 5 s wait period followed by 5 s measurements taken at 7, 1 mm intervals with data acquisition and sensor positioning controlled by the Sensortrace Pro 2.0 (Unisense A/S) dedicated software. Mean experimental N_2_O concentration was calculated from triplicates of three individual experiments. The final mean was corrected for the background signal detected in pure LB.

### Statistical analysis

Data was analyzed for statistical significance with Prism 4 software (GraphPad Software, Inc., La Jolla, CA, USA). Unless otherwise mentioned, parametric data was analyzed by One-Way ANOVA with Dunnett's multiple comparison test and non-parametric by Friedman's test with Dunn's multiple comparison. *P* < 0.05 was considered significant.

## Results

### Denitrification has a significant role in anoxic growth of PAO1 and clinical isolates at physiological levels of NO^−^_3_

**Figure 2:**
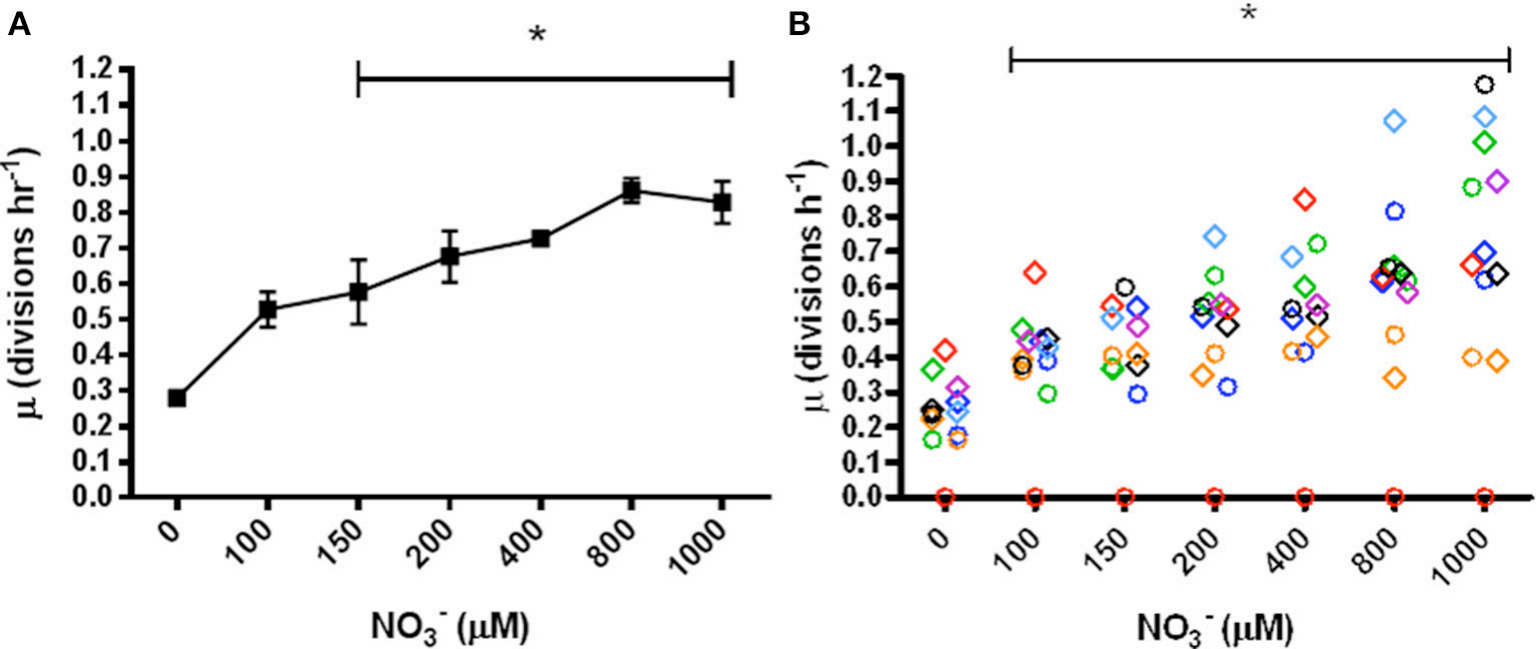
**Specific growth rates (μ) of *P. aeruginosa* in anoxic LB supplemented with NO^−^_3_. (A)** PAO1, mean ± s.e.m. of triplicate experiments, **(B)** clinical isolates, *N* = 12, 7 non-mucoid strains indicated by diamonds and 5 mucoid strains indicated by circles. Colors indicate the 7 patients isolated from ^*^p ≤ 0.05 significant change from 0 μM.

The specific growth rates of *P. aeruginosa* grown for 20 h in anoxic LB supplemented with physiologically relevant concentrations of NO^−^_3_ (0–1 mM of NO^−^_3_) were calculated. PAO1 (Figure 2A) and 12 clinical isolates (5 mucoid and 7 non-mucoid) (Figure 2B) displayed a significant increase in specific growth rate as compared to the unsupplemented control at ≥150 μM NO^−^_3_ (*p* = 0.0138) and ≥100 μM (*p* = 0.0132) NO^−^_3_, respectively. Despite the considerable variation between the growth rates of the clinical isolates, there was no significant correlation with the duration of infection as well as no significant difference between the growth rates of the mucoid and non-mucoid clinical isolates.

The suitability of the assay was demonstrated in a pilot study with anoxic cultures of PAO1 supplemented with 100 mM NO^−^_3_, where our measured growth rate of 0.7 divisions h^−1^ and final OD_600_ of 1.0 (data not shown) corresponded well with previously reported growth rates of *P. aeruginosa* (Alvarez-Ortega and Harwood, 2007; Lee et al., 2012; Fang et al., 2013).

**Figure 3:**
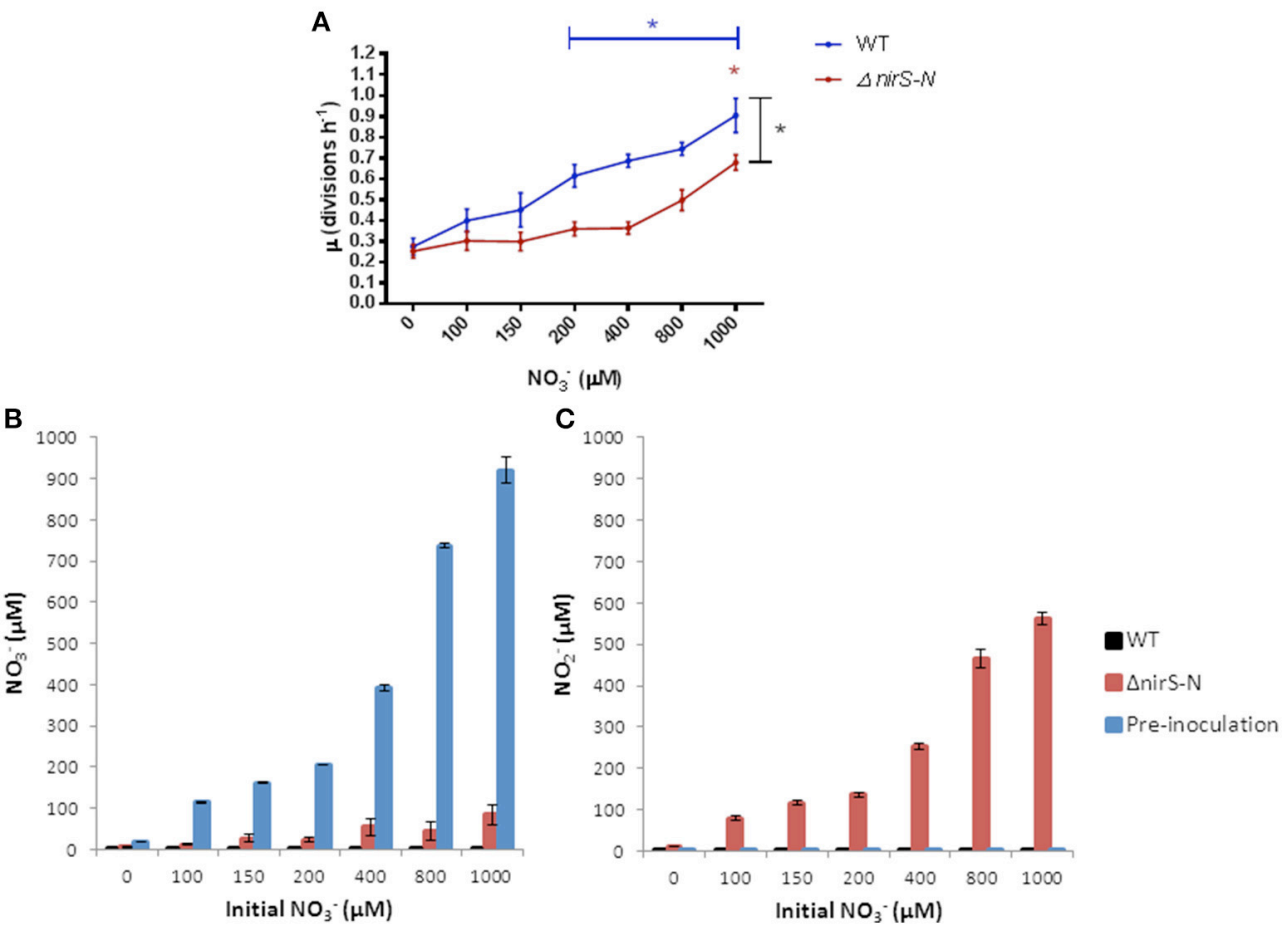
**(A)** Specific growth rates (μ) of *P. aeruginosa* (WT and Δ*nirS-N* PAO1) grown in anoxic LB supplemented with NO^−^_3_. Mean ± s.e.m. of triplicate experiments, ^*^p ≤ 0.05 Blue and red ^*^ indicate significant increase in μ from 0 μM calculated by One-Way ANOVA. Black ^*^ indicate significant difference (*p* = 0.0316) between conditions (≥400 μM) as calculated by Two-Way ANOVA. **(B)** NO^−^_3_ and **(C)** NO^−^_2_ content of anoxic sterile filtered growth media after 20 h.

To discern the portion of growth attributable to denitrification, we compared the specific growth rates in anoxic cultures supplemented with NO^−^_3_ of the WT to a Δ*nirS-N* mutant, which is able to reduce NO^−^_3_ to NO^−^_2_, but whith a blocked first step of strict denitrification due to the inability to reduce NO^−^_2_ to NO, under the same growth conditions were estimated (Figure 3A). NO^−^_3_ supports modest growth of the Δ*nirS-N* mutant with increasing NO^−^_3_ supplementation in accordance with Nar's contribution to proton motive force generation (Chen and Strous, 2013), but with a significant reduction in growth as compared to the WT at NO^−^_3_ ≥400 μM (*p* = 0.0316). One mM NO^−^_3_ was required to initiate a significant increase in growth rate from the unsupplemented control in the Δ*nirS-N* mutant (*p* = 0.0021).

The consumption of NO^−^_3_ and the accumulation of NO^−^_2_ in the sterile filtered growth media of the Δ*nirS-N* mutant at 20 h confirmed that the ability to reduce NO^−^_3_ to NO^−^_2_ was present in the Δ*nirS-N* mutant, but that the mutant failed to further reduce NO^−^_2_ as opposed to the total consumption of NO^−^_2_ by the WT. Furthemore, we demonstrated very low concentrations of NO^−^_3_ and NO^−^_2_ pre-inoculation in LB and that the concentration of NO^−^_2_ was not affected by NO^−^_3_ (Figures 3B,C).

### Dynamic increase in denitrification reductase promoter activation at 400 μM NO^−^_3_

**Figure 4:**
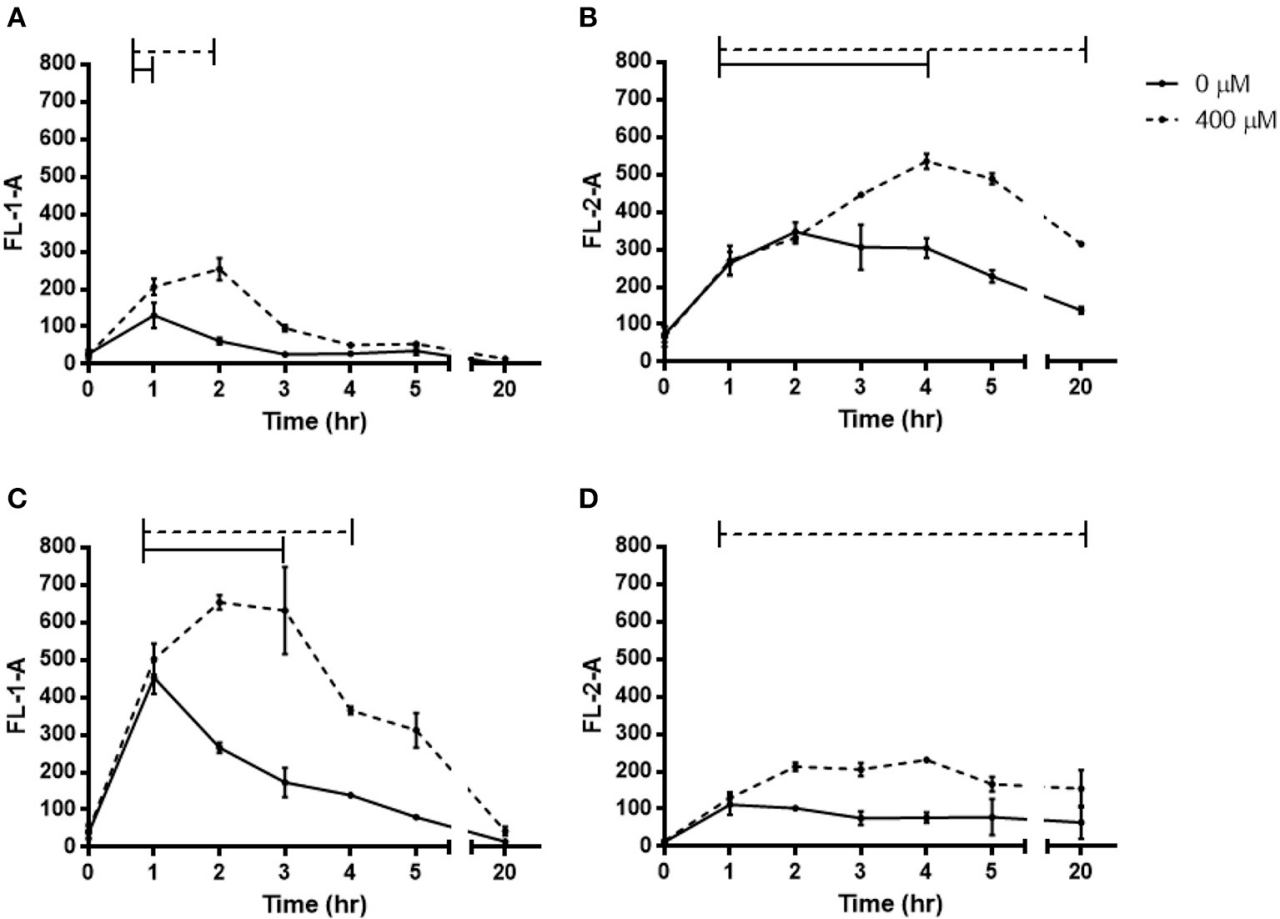
**Fluorescence of denitrification reporter promoters of *P. aeruginosa* PAO1 during anoxic growth with 0 or 400 μM NO^−^_3_. (A)** *narK_1_-eGFP*, **(B)** *nirS-dsredexpress*, **(C)** *norC-eGFP*, **(D)** *nosZ-dsredexpress*. Mean ± s.e.m. as measured by flow cytometry from triplicates. eGFP was detected in FL-1-A, DsRed-Express was detected in FL-2-A. Bars above represent the period of time of significant increase *p* ≤ 0.05.

**Figure 5:**
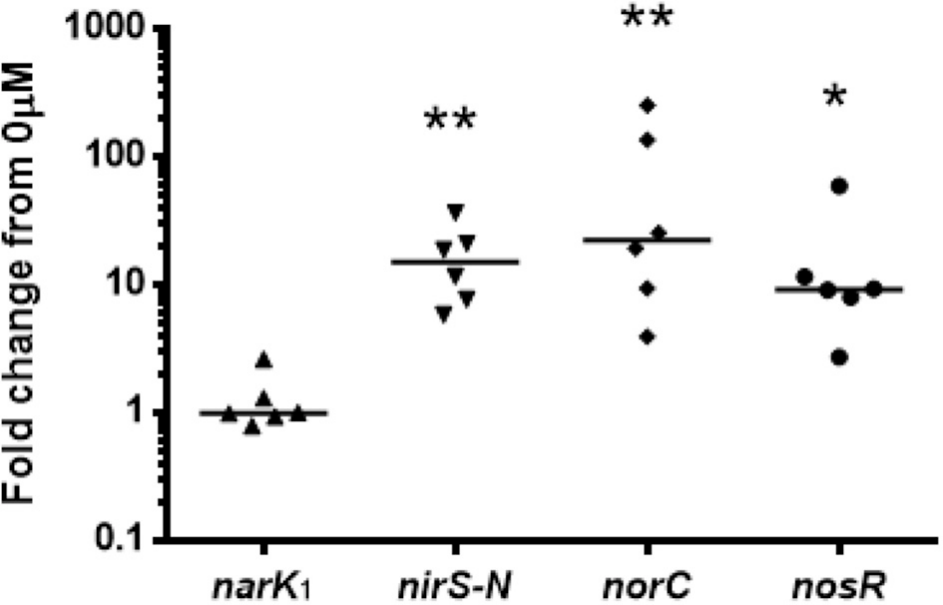
**qRT-PCR on denitrification reductase genes of *P. aeruginosa* PAO1 under anoxic growth for 3 h in LB with 0 or 400 μM NO^−^_3_**. Expression was normalized to the housekeeping gene *rpoD*. Values show fold change from 0 μM with theoretical median of 1. *N* = 6 with median. ^*^p ≤ 0.05, ^**^p ≤ 0.01.

The reporter strains were grown under anoxia for 20 h supplemented with 400 μM NO^−^_3_ and the fluorescence measured post chloramphenicol addition with OFR by flow cytometry (Figure 4). The activity of all four promoters was significantly increased during supplementation with 400 μM NO_3_, but the time course varied. The Nar activity showed a small peak at 2 h (*p* < 0.0001) in comparison to Nor, which showed a stronger peak at 2–3 h (*p* < 0.0001). Nir and Nos first showed significant upregulated promoter activity after 1 h and continued to be so over 20 h (*p* < 0.0001 and 0.0273). In all reductases other than the Nos, there were also periods of significant upregulation in the unsupplemented control, but for shorter periods of time and with lower peaks. The transcripts of the denitrification reductase genes in PAO1 grown under anoxia with 400 μM NO^−^_3_ hours showed significant upregulation of *nirS, norC* and *nosR* after 3 h (*p*-values = 0.014, 0.014, and 0.04) (Figure 5).

We noted that anoxic conditions alone were apparently sufficient to trigger upregulation of some denitrification reductases as previously observed in other planktonic and biofilm studies (Härtig and Zumft, 1999; Hentzer et al., 2005; Schreiber et al., 2007) and that LB is a rich medium which we found to contain ~20 μM NO^−^_3_. We speculate that this is an explanation for the observed upregulation in our unsupplemented controls. However, the focus of the present study is on the differences due to the additional physiological levels of NO^−^_3_ supplementation.

### NO^−^_3_ is rapidly consumed with a subsequent transient increase of NO^−^_2_ in anoxic growth media

**Figure 6:**
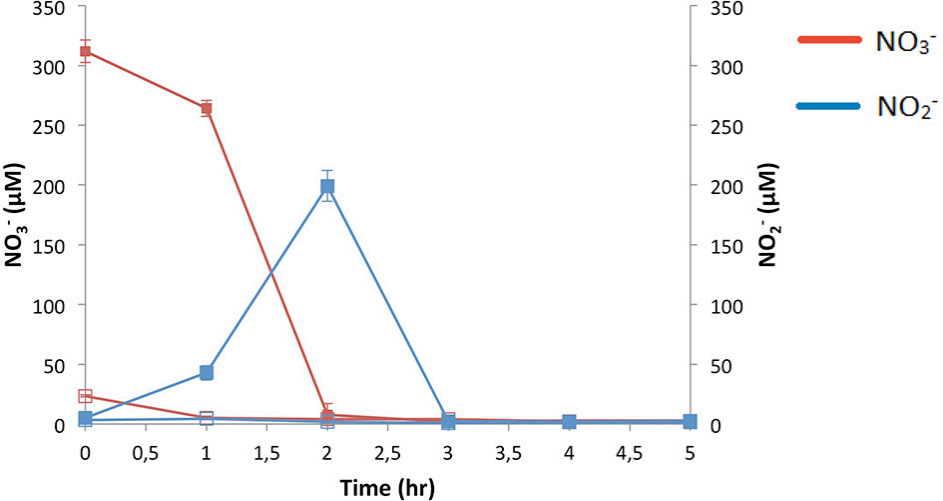
**Consumption and production of NO^−^_3_ and NO^−^_2_ during anaerobic growth of *P. aeruginosa* PAO1**. *N* = 4, mean ± s.e.m. Initial NO^−^_3_: Open squares 0 μM KNO_3_, closed squares 400 μM KNO_3_.

Medium from the reporter PAO1 strains showed that 400 μM NO^−^_3_ was depleted to below the detection level of the Griess assay within just 2 h with a transient increase in the product of its reduction, NO^−^_2_ (Figure 6). This rapid depletion combined with the time taken to harvest the supernatant is the likely explanation for the initial time point showing somewhat less than the expected 400 μM NO^−^_3_, as the media pre-inoculation was tested and confirmed to contain 400 μM NO^−^_3_.

### N_2_O is produced in response to NO^−^_3_ supplementation

**Figure 7:**
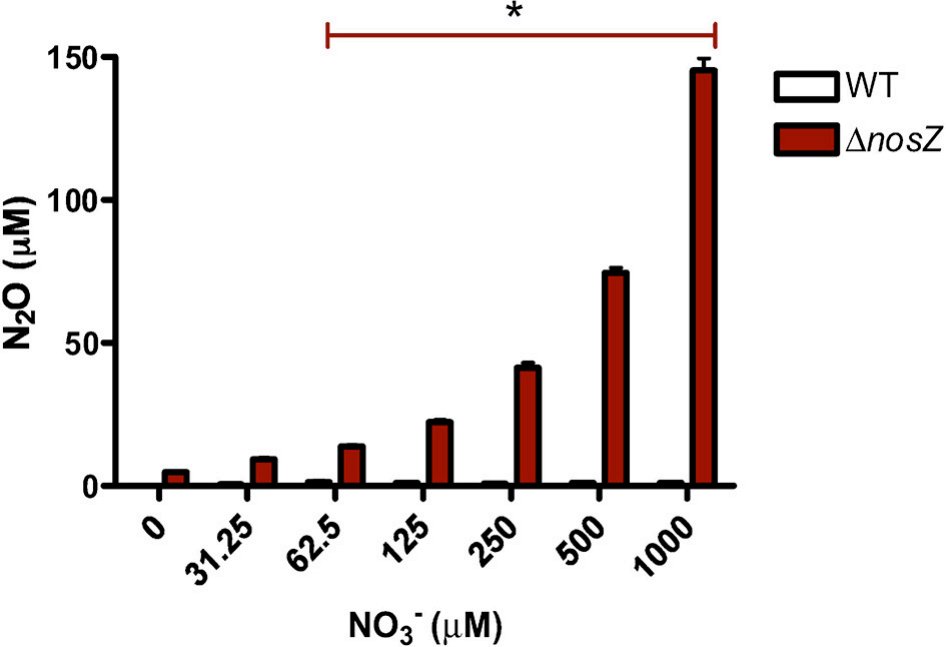
**N_2_O production by *P. aeruginosa* PAO1 grown over 20 h in anoxic LB supplemented with NO^−^_3_ in triplicate**. Mean ± s.e.m. ^*^p ≤ 0.05 indicating significant increase of N_2_O production by Δ*nosZ*.

The Δ*nosZ* mutant and a WT PAO1 strain were grown with physiological concentrations of NO^−^_3_ under anoxia and the average N_2_O accumulation over 20 h detected. With as little as 62.5 μM NO^−^_3_, a significant production of N_2_O was stimulated (*p* = 0.0125) (Figure 7). The WT produced <10 μM of N_2_O at all experimental concentrations of NO^−^_3_; confirming the defective conversion of N_2_O to N_2_ by the Δ*nosZ* mutant.

## Discussion

*P. aeruginosa* is able to thrive under anoxic conditions by performing denitrification. Our initial studies performed with clinical isolates and PAO1 under anoxic conditions showed that physiological concentrations of NO^−^_3_ are sufficient to support a significant increase in growth rate, even in cultures supplemented with less NO^−^_3_ than the 400 μM applied by Palmer et al. (2007).

The anoxic growth rates obtained are comparable to those reported in CF lungs and sputum, except for the very low growth rates in some of the clinical samples from CF lungs and sputum (Yang et al., 2008; Kragh et al., 2014). This is likely due to our planktonic studies representing a slightly faster growth rate as the shaken cells have equal access to nutrients in the media, whereas in the biofilm mode of growth in the lungs, internal cells are assumed to have access to less nutrients, including NO^−^_3_, due to diffusion limitation and utilization by cells at the periphery of the biofilm (Bjarnsholt et al., 2009). Additionally, ~10% of the cells in clinical samples appear to be in stationary phase (Yang et al., 2008) whereas the growth rates in our experiments are calculated from the exponential growth phase. In the lungs, *P. aeruginosa* is also under continual attack from the host inflammatory response and antibiotic stress (Høiby, 2006), which is also likely to reduce the rate of growth.

Nar is required for anoxic growth at physiological levels of NO^−^_3_ in CF sputum (Palmer et al., 2007) and accordingly NO^−^_3_ reduction alone is able to generate some growth as seen in the Δ*nirS-N* mutant, unable to perform the subsequent NO^−^_2_ reduction and therefore blocking denitrification. However, we demonstrate that the Δ*nirS-N* mutant requires the highest physiological concentration of 1 mM NO^−^_3_, seen only in exacerbated CF patients following antimicrobial treatment (Grasemann et al., 1998), to enable a significant increase in growth rate as well as a significant reduction of growth as compared to the WT. This indicates a substantial additional contribution of denitrification to the observed anoxic growth of *P. aeruginosa* at physiological levels of NO^−^_3_. Anoxic growth of mutants with defective Nir was also reduced at concentrations of NO^−^_3_ greater than physiological levels [10 mM (Schreiber et al., 2007) and 100 mM (Filiatrault et al., 2006)].

In addition to supporting anoxic growth, Nir may promote the persistence of *P. aeruginosa* in the infected CF lungs as Nir is required for type III secretion resulting in prolonged survival in human monocytes (Van Alst et al., 2009) and enhanced virulence (Filiatrault et al., 2006). This supports the idea that denitrification is necessary for achieving the observed growth of *P. aeruginosa* in the lungs.

The dynamic increase in the promoter activity of all four denitrification reductases over 20 h and increased expression of all transcripts but the *narK_1_* at 3 h with 400 μM NO^−^_3_ further emphasized the engagement of denitrification. The explanation for the transient peak in Nar expression and lack of upregulation at 3 h became apparent after analysing the growth media for NO^−^_3_ content, showing depletion after just 2 h. Expression of Nar is controlled in part by the two-component NO^−^_3_-sensing regulator NarX-L (Schreiber et al., 2007; Schobert and Jahn, 2010) so presumably after NO^−^_3_ depletion Nar expression is no longer induced. The Nor is also proposed to be under NarX-L control (Schobert and Jahn, 2010), which would explain the decrease in promoter activity after 2 h.

The Nar product, NO^−^_2_ displayed a transient increase in the growth media which was similarly rapidly depleted by 3 h. However, the expression of Nir and Nos appeared to be less tightly regulated by N-oxide concentration as they were still significantly upregulated at 20 h, and in the case of the Nir, only a modest increase in expression was stimulated by NO^−^_3_ supplementation. The additional O_2_-sensing Anr regulator and downstream NO sensing dissimilative nitrate respiration (Dnr) regulator (Schreiber et al., 2007; Schobert and Jahn, 2010; Trunk et al., 2010; Arai, 2011) may play a more important role in Nir and Nos regulation (Schreiber et al., 2007; Schobert and Jahn, 2010) and hence their expression more responsive to the anoxic conditions and the downstream denitrification product NO resulting in prolonged increase in expression.

As N-oxides are not exclusively utilized in strict denitrification—NO^−^_3_ reduction is also able to feed into dissimilatory pathways for ammonia excretion or into assimilatory pathways for incorporation into biomolecules (Zumft, 1997)—we also studied N_2_O production. The known pathways for N_2_O production are denitrification and nitrification via oxidation of hydroxylamine to N_2_O (Khalila et al., 2004). As of yet, there is no evidence to suggest that *P. aeruginosa* performs or possesses homologs of the enzymes required for nitrification [PAO1 Genome Sequence, Pseudomonas Genome Database (Winsor et al., 2011)]. Additionally, the process requires O_2_ (Meyer et al., 2008) and thus for our anoxic experiments we assumed that all detected N_2_O was produced via the denitrification pathway and therefore acted as a specific marker. In the *P. aeruginosa* nitrous oxide reductase mutant Δ*nosZ*, unableto reduce N_2_O to N_2_, we found a significant accumulation of N_2_O in the anoxic growth media with as little NO^−^_3_ supplementation as 62.5 μM. This finding further supports that *P. aeruginosa* employs denitrification to obtain energy for growth at physiological levels of NO^−^_3_ in anoxic conditions.

Interestingly, one late clinical strain isolated from a patient chronically infected for 35 years, was unable to grow in anoxic conditions with NO^−^_3_. This may relate to adaptations of more flexible nitrate metabolism over time, with denitrification suggested to no longer be essential for growth for some strains at later phases of infection (Oberhardt et al., 2010; Cramer et al., 2011). While NO^−^_3_ was unable to stimulate anoxic growth of the late isolate, alternative anoxic growth may occur by arginine fermentation (Vander Wauven et al., 1984) and survival could be facilitated by pyruvate fermentation (Eschbach et al., 2004) and phenazine redox cycling (Glasser et al., 2014; Quinn et al., 2014). Alternatively, the late isolate may have adapted to favor growth in the alveoles where oxygen is supplied by inspired air and capillaries surrounding the alveoles (West, 2000).

It has previously been shown that micro-oxic conditions are able to support growth of *P. aeruginosa* (Alvarez-Ortega and Harwood, 2007). Here we substantiate that physiological concentrations of NO^−^_3_ are also able to support growth by denitrification in anoxic conditions, highlighting the flexibility of *P. aeruginosa's* ability to survive in a range of environments in the CF airways (Worlitzsch et al., 2002; Aanaes et al., 2011; Kolpen et al., 2014a). Interestingly, physiological concentrations of NO^−^_3_ may also enhance growth of *P. aeruginosa* even at micro-oxic conditions since a growth advantage of laboratory strains carrying a *lasR* mutation, which is associated with severe lung damage in infected CF patients (Hoffman et al., 2009), was obtained with supplementation of only 125 μM NO^−^_3_ in conditions with limited O_2_ mass-transfer (Hoffman et al., 2010).

In conclusion, *P. aeruginosa* is readily able to adapt to the physiological levels of NO^−^_3_ and lack of O_2_ in the infected CF lungs by employing denitrification. The ability of *P. aeruginosa* to grow under anoxia has clinical relevance as multiple classes of antibiotics are known to have reduced efficacy in the absence of O_2_ (Walters et al., 2003; Dwyer et al., 2014). Denitrification may consequently be a major factor in *P. aeruginosa's* observed tolerance to antibiotic treatment in CF patients (Høiby et al., 2010). This tolerance may be overcome by directing antibacterial treatment against denitrification as suggested by the downregulation of *narG* and *narH* resulting in increased susceptibility of *P. aeruginosa* to fosfomycin and tobramycin combination treatment under anoxic conditions (McCaughey et al., 2013). Denitrification has also recently been proposed to contribute to the pathogenicity of bacterial species infecting CF lungs due to its activity in *P. aeruginosa* as well as the highly pathogenic species *Achromobacter xylosoxidans* and *Burkholderia multivorans* in comparison to the less pathogenic *Stenotrophomonas maltophilia* which is unable to perform denitrification (Kolpen et al., 2014c). Accordingly, our results underline the importance of developing novel treatments that target *P. aeruginosa's* flexible metabolism more broadly, with the denitrification reductases presenting potential targets, and that can be administered to reach the varied lung microenvironments that *P. aeruginosa* is able to adapt to and persist within.

### Conflict of interest statement

The authors declare that the research was conducted in the absence of any commercial or financial relationships that could be construed as a potential conflict of interest.
